# PTEN Inhibition Protects Against Experimental Intracerebral Hemorrhage-Induced Brain Injury Through PTEN/E2F1/β-Catenin Pathway

**DOI:** 10.3389/fnmol.2019.00281

**Published:** 2019-12-05

**Authors:** Dan Zhao, Xing-Ping Qin, Song-Feng Chen, Xin-Yu Liao, Jing Cheng, Rui Liu, Yang Lei, Zhi-Feng Zhang, Qi Wan

**Affiliations:** ^1^Department of Physiology, School of Basic Medical Sciences, Hubei University of Medicine, Shiyan, China; ^2^Department of Neurosurgery, Renmin Hospital of Wuhan University, Wuhan, China; ^3^Department of Physiology, Collaborative Innovation Center for Brain Science, School of Basic Medical Sciences, Wuhan University School of Medicine, Wuhan, China; ^4^Institute of Neuroregeneration and Neurorehabilitation, Department of Neurosurgery of the Affiliated Hospital, Qingdao University, Qingdao, China

**Keywords:** intracerebral hemorrhage, PTEN, bpV[pic], E2F1, β-catenin, neuroprotection

## Abstract

Intracerebral hemorrhage (ICH) is a subtype of stroke with highest mortality and morbidity. We have previously demonstrated that dipotassium bisperoxo (picolinato) oxovanadate (V), (bpV[pic]) inhibits phosphatase and tensin homolog (PTEN) and activates extracellular signal-regulated kinase (ERK)1/2. In this study, we examined the effect of bpV[pic] in the rat ICH model *in vivo* and the hemin-induced injury model in rat cortical cultures. The rat model of ICH was created by injecting autologous blood into the striatum, and bpV[pic] was intraperitoneally injected. The effects of bpV[pic] were evaluated by neurological tests, Fluoro-Jade C (FJC) staining, and Nissl staining. We demonstrate that bpV[pic] attenuates ICH-induced brain injury *in vivo* and hemin-induced neuron injury *in vitro*. The expression of E2F1 was increased, but β-catenin expression was decreased after ICH, and the altered expressions of E2F1 and β-catenin after ICH were blocked by bpV[pic] treatment. Our results further show that bpV[pic] increases β-catenin expression through downregulating E2F1 in cortical neurons and prevents hemin-induced neuronal damage through E2F1 downregulation and subsequent upregulation of β-catenin. By testing the effect of PTEN-siRNA, PTEN cDNA, or combined use of ERK1/2 inhibitor and bpV[pic] in cultured cortical neurons after hemin-induced injury, we provide evidence suggesting that PTEN inhibition by bpV[pic] confers neuroprotection through E2F1 and β-catenin pathway, but the neuroprotective role of ERK1/2 activation by bpV[pic] cannot be excluded.

## Introduction

Intracerebral hemorrhage (ICH) is a subtype of stroke that is caused by rupture of blood vessels in the brain parenchyma usually due to hypertension; 40–50% of patients died within the first 30 days (Flower and Smith, 2011; Howitt et al., 2012). Despite extensive researches, there is no effective treatment to improve the chances of survival in patients with ICH (Zheng et al., 2016). Phosphatase and tensin homolog (PTEN) is a dual phosphatase that not only dephosphorylates the lipid substrate but also dephosphorylates the protein substrate (Schmid et al., 2004). Our previous studies show that inhibiting PTEN, through enhancing γ-aminobutyric acid (GABA)_A_ receptor expression and function, protects against neuronal death both *in vitro* and *in vivo* (Liu et al., 2010). We and others also provide evidence indicating that inhibiting the function of lipid phosphatase of PTEN is neuroprotective after ischemia-reperfusion injury (Ning et al., 2004; Chang et al., 2007; Zhang et al., 2007; Zheng et al., 2012). However, the role of PTEN inhibition in ICH injury is unknown.

The transcription factor E2F transcription factor 1 (E2F1) is a key regulator of cell cycle, which is essential for cell apoptosis and proliferation (Hallstrom et al., 2008; Poppy Roworth et al., 2015; Denechaud et al., 2016; Shats et al., 2017). E2F1-mediated apoptotic program was blocked by the best known phosphoinositide 3-kinase (PI3K)/protein kinase B (Akt) signaling, which was negatively regulated by PTEN. Thus, PTEN inhibition promotes Akt-dependent cell survival (Maehama and Dixon, 1998; Yamada and Araki, 2001; Hallstrom et al., 2008; Milella et al., 2015). It is reported that cyclin-dependent kinase (CDK) inhibitor blocks the increase of E2F1 level and reduces neuronal death in ischemic stroke (Osuga et al., 2000). Also, evidence suggests that absence of E2F1 attenuates brain damage and improves postischemic behavior in mice (MacManus et al., 2003). The E2F1-deficient mice suffer less ischemic damage after 24 h reperfusion, which suggests that E2F1 plays a critical role in promoting cell death in brain ischemia (MacManus et al., 1999). In addition, a recent study shows that PTEN binds to and interacts with the E2F1 promoter region, thus regulating E2F1-mediated transcription in lung cancer (Malaney et al., 2018). Together, these studies lead us to reason that downregulation of E2F1 by PTEN inhibition may play a neuroprotective role in ICH injury.

β-catenin is a part of cadherin protein complex, which acts as a signal transducer in the Wnt/β-catenin pathway (Maeda et al., 2013). Recent studies show that β-catenin plays an important role in mitochondrial homeostasis under pathophysiological conditions (Hsu et al., 2014). Activation of Wnt/β-catenin signaling alleviates the disruption of blood-brain barrier (BBB) and the hemorrhage defects in Gpr124-CKO mice (Chang et al., 2017). Substantial evidences suggest that the β-catenin pathway is a key pathway in regulating neurogenesis (Hussaini et al., 2014; Tiwari et al., 2014). Activation of β-catenin inhibits prion protein-induced apoptosis to exert a neuroprotective effect (Jeong et al., 2014). It is reported that β-catenin is regulated by E2F1 (Morris et al., 2008). E2F1 suppresses β-catenin activity and reduces the expression of β-catenin targets including survivin and c-MYC (Morris et al., 2008). Together, these results indicate that activation of β-catenin signaling confers neuroprotection.

In this study, we investigate the relationship between PTEN, E2F1, and β-catenin in a rat model of ICH injury. We demonstrate that PTEN inhibition protects against ICH-induced brain injury *via* PTEN/E2F1/β-catenin signal pathway, which may serve as potential therapeutic targets of ICH therapy.

## Materials and Methods

### Animals and ICH Model

All procedures were conducted following an institutionally approved protocol in accordance with the National Institutes of Health Guide for the Care and Use of Laboratory Animals. Male rats (*n* = 328, weighing 280–300 g) were housed in a light-and temperature-controlled environment and supplied with adequate food and water. Randomization is used to assign samples to the experimental group and collect and process the data. Experiments were conducted by researchers blinded to the group assigned to each animal. All studies involving animals are reported in accordance with the ARRIVE guidelines for reporting experiments involving animals. ICH mice were induced using a modified double infusion model of autologous whole blood (100 μl) as previously reported (Chang et al., 2017; Jiang et al., 2017). Briefly, mice were anesthetized *via* intraperitoneal 10% hydrate (3.5 ml/kg). Positioned prone onto a stereotactic head frame (Shenzhen Wo Ruide Life Technology Co.), a craniotomy was performed, and a 27-gauge needle was inserted into the left striatum (stereotactic coordinates from Bregma: 0.2 mm anterior, 3.5 mm lateral, and 5.5 mm in depth). Autologous whole blood (100 μl), which was collected from the femoral artery, was infused at a rate of 10 μl/min. The needle was then lowered to a target position with a depth of 5.5 mm. After 5 min, 100 μl of autologous whole blood was injected into the left striatum. When the injection was completed, the needle was left in place for 10 min and then removed at a rate of 1 mm/min. The craniotomy was sealed with bone wax, and the scalp suture was closed. Sham-operated animals only underwent needle insertion. The core body temperature was maintained at 37°C ± 0.5 during the entire procedure and for 2 h after the surgery.

### Drug Administration

Animals were given dipotassium bisperoxo (picolinato) oxovanadate (V), (bpV[pic]; Santa Cruz Biotechnology, Santa Cruz, CA, USA) at a dose of 20 μg/kg, 0.2 mg/kg, 2 mg/kg, or vehicle (saline) 0.5 h after ICH by intraperitoneal injection three times in total with an interval of 4 h. The dose regimen is based on previous studies (Sury et al., 2011). The E2F1 inhibitor HLM006474 (25 μM, 2 μl; Merck-Millipore, Germany) and β-catenin inhibitor ICG-001 (50 mM, 2 μl; Axon Medchem, Groningen, the Netherlands) were injected into the left lateral ventricle (from the Bregma: 0.8 mm posterior, 1.5 mm lateral, and 3.5 mm deep) with a Hamilton micro syringe at a rate of 1 μl/min 0.5 h after ICH while the rats were under anesthesia. The animals were randomly assigned to experimental groups, and the experimenters were blinded to group assignment. For hemin-induced neurotoxicity studies, we treated the cultures with bpV[pic] (20, 50, 100, 200 nM) for 6 h at 30 min after hemin injection (Liu et al., 2010).

### Western Blot

Rats were sacrificed, and the brains were removed for Western blot test. Peripheral tissues surrounding the hematoma were homogenized in RIPA buffer for 30 min on ice using a tissue grinder. Tissue lysates were then centrifuged at 12,000× *g* for 15 min at 4°C, and total protein concentration was assessed using the BCA Protein Assay Kit. For the detection of PTEN and other proteins, the samples prepared in the same day were used. The polyvinylidene difluoride membrane (Millipore, Bedford, MA, USA) was incubated with primary antibody against PTEN (1:1,000; Cell Signaling Technology, Beverly, MA, USA), E2F1 (1:500; Abcam), β-catenin (1:1,000; Abcam), survivin (1:500; Santa Cruz Biotechnology, Dallas, TX, USA), β-actin (1:2,000; Santa Cruz Biotechnology, Dallas, TX, USA). The primary antibodies were labeled with a horseradish peroxidase-conjugated secondary antibody, and the protein bands were imaged using a Super Signal West Femto Maximum Sensitivity Substrate (Pierce, Rockford, IL, USA). Blot images were obtained directly from the polyvinylidene difluoride membrane using an EC3 imaging system (UVP, LLC, Upland, CA, USA). ImageJ software was used to quantify the Western blot data.

### Immunofluorescent Staining

Rats were sacrificed 24 h after ICH, perfused with iced phosphate-buffered saline (PBS), followed by 4% PFA. Then, their brains were removed and fixed with 4% PFA/30% sucrose. The tissue was then frozen in Tissue-Tek OCT mounting medium, and 18-μm coronal sections were cut. Brain sections were incubated at 4°C overnight with PTEN primary antibody (1:200; Cell Signaling Technology, Danvers, MA, USA), E2F1 primary antibody (1:200; Abcam), or β-catenin primary antibody (1:200; Abcam) diluted in a blocking solution of 1% bovine serum albumin (BSA) and 0.1% Triton X-100. The tissues were then incubated with a secondary antibody (Alexa-594 conjugated to goat anti-mouse) or a secondary antibody (Alexa-594 conjugated to goat anti-rat) for 1 h at 37°C. Then the tissue was incubated at 37°C for 3 h with primary antibody of NeuN (1:200; Millipore, Burlington, MA, USA) followed with secondary antibody (Alexa-488 conjugated to goat anti-mouse), washed three times with PBS, and observed under an Olympus fluorescent microscope (IX51, Olympus).

### Fluoro-Jade C (FJC) Staining

FJC staining is well-known to assess the degenerating neurons, as previously described (Xie et al., 2017). Procedure was conducted as the study reported (Kononenko et al., 2017). Briefly, frozen sections were mounted on gelatin-coated slides and air-dried overnight. Slides were incubated in 0.06% potassium permanganate and then incubated in 0.0001% FJC (Histochem, Jefferson, AR, USA). Tissue was then dried in an oven at 60°C and incubated in xylene for 1 min, then coverslips were mounted with cover glass. For each brain, three slices between Bregma −2.5 and −1.5 were imaged for quantification. FJC-positive cells were counted by blinded observers using ImageJ. The average number of FJC-positive cells per section was reported.

### Cortical Neuron Culture, Transfection, and Hemin Administration

The cortical neuronal cultures were prepared from Sprague–Dawley rats at gestation day 17 as described (Shan et al., 2009; Zhang et al., 2017). Briefly, the isolated cortical neurons were suspended in the inoculation medium (Neurobasal medium, 0.5% FBS, 2% B 27 supplement, 0.5 μM L-glutamine, 25 μM glutamic acid). Then, the neurons were plated in a Petri dish coated with D-lysine in advance. One day later, half of the inoculation medium was removed, and half of the serum-free medium (Neurobasal medium, 2% B-27 supplement and 0.5 μM L-glutamine) was added. The serum-free medium was then changed every 3 days. By 12 days, immunofluorescence staining was performed, and neurons within less than 2% glial cells were used for experimental detection. The PTEN overexpression vector was constructed by Genechem (Shanghai, China, GOSE91553) as in our previous study (Chen et al., 2016). PTEN siRNA (sc-29459), E2F1 siRNA (sc-29297), and control siRNA (sc-44236) were purchased from Santa Cruz Biotechnology. The treatment was performed in cultured cortical neurons based on the manufacturer’s instructions. After a 48-h culture, knockdown efficiency was typically measured at the protein level by Western blot or immunofluorescent staining. NsiRNA-negative control was used as a control. To simulate experimental ICH, the cultures were exposed to various concentrations (50, 100, and 200 μM) of hemin (Sigma) or vehicle (0.1% dimethyl sulfoxide, DMSO) for 6 h *in vitro* before being harvested for cell survival analyses (He et al., 2010; Lin et al., 2012). The hemin was dissolved in DMSO to prepare a stock solution at the concentration of 50 mM and diluted to the working concentration with fresh medium. The final concentration of DMSO was less than 0.1%.

### Lactate Hydrogenase (LDH) Assay

LDH is released from damaged cells. It was measured according to the CytoTox 96 Cytotoxicity kit instructions. Neurons were treated with 10× lysis solution to completely lyse the cells to measure the maximum release level of LDH. Absorbance values were read at 490 nm using a 96-well plate reader (Molecular Devices, San Jose, CA, USA). According to the manufacturer’s instructions, the LDH release (%) was calculated by calculating the ratio of experimental LDH release to maximal LDH release.

### MTT Assay

In this experiment, the viability of cells was assessed by the ability of cells to take up thiazolyl blue tetrazolium bromide (MTT). Cells were incubated with MTT for 1 h, then lysed with DMSO and waited in the dark overnight at room temperature. The lysate was then read on a plate reader at an absorbance wavelength of 540 nm.

### Behavioral Assessment

Behavioral assessment was conducted at 1 day before surgery and days 0, 1, and 3 after ICH using the modified Neurological Severity Score (mNSS) and corner test to comprehensively evaluate motor, sensory, reflex, and balance functions, as previously described (Ma et al., 2017; Ren et al., 2018; Yang et al., 2018). We performed neurobehavioral tests at 6 h after ICH at day 0 as we planned to observe the possible improvement between day 0 and day 1/3. The mNSS score ranges from 0 to 18. A score of 13–18 indicates severe injury, a score of 7–12 indicates moderate injury, and a score of 1–6 indicates mild injury. If the rats fail to complete the task, 1 point was given. During the corner test, rats were allowed to enter a 30° corner and then they will turn to the right or left. The procedure was repeated 10 times for every rat for at least 30 s between trials. Then the percentage of right turn was calculated.

#### Statistics

The data were analyzed by SPSS 19.0 software and expressed as the mean ± SD. Student’s *t*-test, ANOVA followed by the Tukey *post hoc* test, or two-way ANOVA followed by Bonferroni *post hoc* test was used where appropriate. Values of *p* < 0.05 are considered significant.

## Results

### BpV[pic] Confers Neuroprotection in a Rat Model of ICH

**Figure 1:**
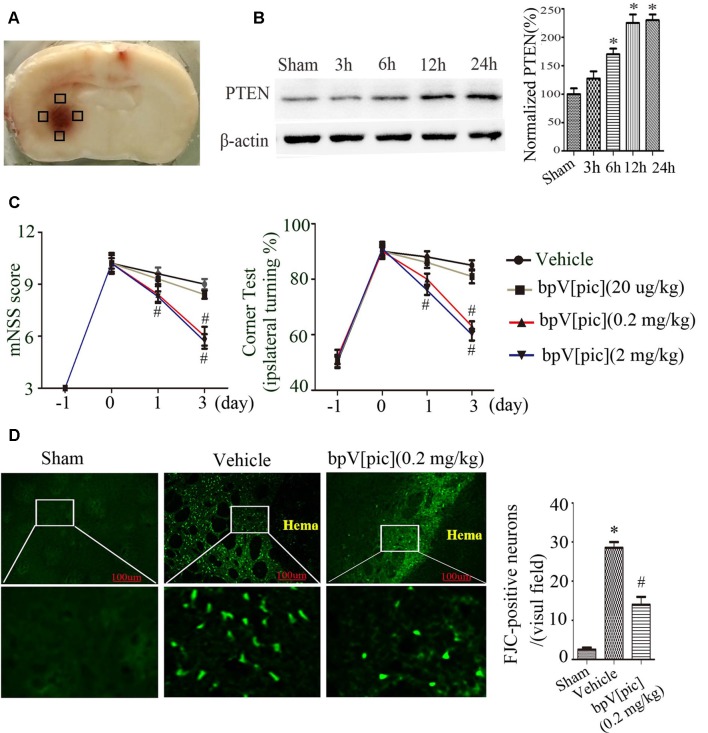
Dipotassium bisperoxo (picolinato) oxovanadate (V), (BpV[pic]) attenuated neurological deficits and decreases neuron loss after intraceberal hemorrhage (ICH) induction. **(A)** Represents the slice of rat brain 24 h after experimental ICH. Black box represents the four sites around the hematoma of the Western blot experiment. **(B)** Expression of phosphatase and tensin homolog (PTEN) at different time points after rat ICH. **(C)** Neurological tests were performed in rats receiving vehicle or bpV[pic] (20 μg/kg, 0.2 mg/kg, 2 mg/kg). **(D)** Rats received vehicle or bpV[pic] at a dose of 0.2 mg/kg 0.5 h after ICH by intraperitoneal injection. Fluoro-Jade C (FJC)-positive neurons were tested in the peri-hematoma 24 h after ICH (*p < 0.05 compared to Sham, ^#^*p* < 0.05 compared to Vehicle. One-way ANOVA test, followed by Bonferroni *post hoc* test, *N = 8*).

We first set up to determine the effect of PTEN inhibition using PTEN inhibitor bpV[pic] in rat model of ICH injury. Figure 1A shows the hematoma after rat ICH induction. Our results show that the expression of PTEN in peri-hematomal area was increased at the early times (3–12 h after ICH; Figure 1B). We tested the effect of bpV[pic] in ICH injury by examining the neurological deficits, neuronal death, and neuronal loss in ICH rats. ICH rats received an injection of bpV[pic] (20 μg/kg, 0.2 mg/kg, 2 mg/kg) or vehicle three times every 4 h starting 0.5 h after ICH induction (Sury et al., 2011). Neurological function was evaluated by performing mNSS and corner tests at days 0, 1, and 3 after ICH. FJC and Nissl staining were conducted at 24 h after ICH. Compared with vehicle recipients, bpV[pic] (0.2, 2 mg/kg)-treated mice had significant reduction of neurological deficits after ICH (Figure 1C). The administration of bpV[pic] (0.2 mg/kg) reduced the number of positive FJC neurons (Figure 1D). Nissl staining also shows that bpV[pic] (0.2 mg/kg) protects against ICH-induced neuron loss (Supplementary Figure S1).

### BpV[pic] Treatment Prevents the Altered Expression of E2F1 and β-Catenin After ICH

**Figure 2:**
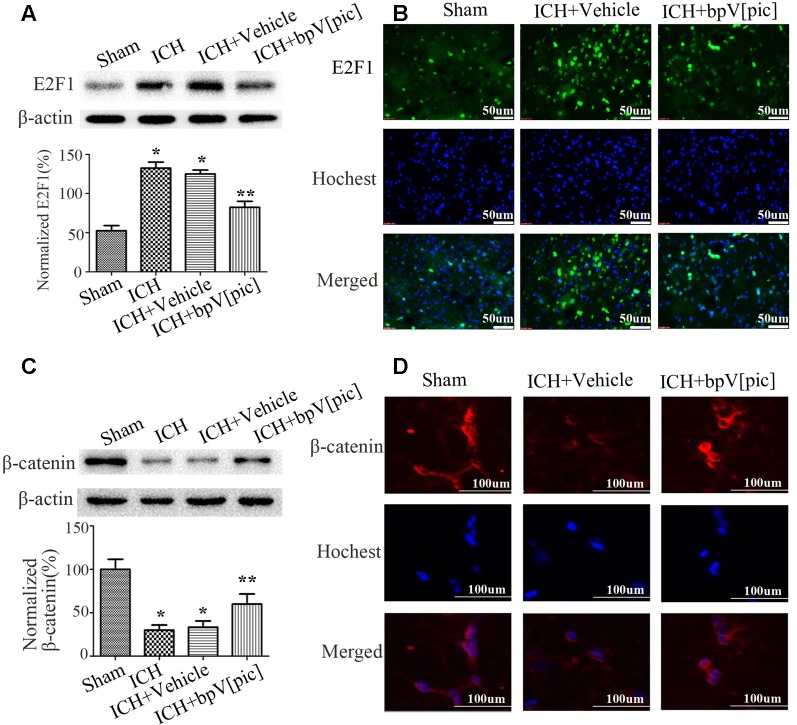
BpV[pic] reversed the changes of E2F1 and β-catenin after rat experimental intracerebral hemorrhage (ICH). **(A,B)** Western bolt or immunofluorescence analysis of E2F1 expression after bpV[pic] treatment at a dose of 0.2 mg/kg 0.5 h after ICH in rats. **(C,D)** Western blot or immunofluorescence analysis of β-catenin expression after bpV[pic] treatment at a dose of 0.2 mg/kg 0.5 h after ICH in rats (*p < 0.05 compared to Sham, **p < 0.05 compared to ICH+Vehicle; one-way ANOVA test, followed by Bonferroni *post hoc* test, *N* = 8).

To explore whether E2F1 and β-catenin signaling is involved in ICH injury, we tested the expression of E2F1 and β-catenin after ICH. The results of Western blot (Figures 2A,C) and immunofluorescence staining (Figures 2B,D) show that the expression of E2F1 is increased while β-catenin is decreased after ICH injury. To determine whether bpV[pic] protects against neuronal injuries through E2F1 or/and β-catenin signaling in ICH rats, we tested the expression of E2F1 and β-catenin in ICH rats treated with bpV[pic] or vehicle. We show that bpV[pic] administration attenuates the change of E2F1 and β-catenin after ICH induction. These results suggest a possibility that the neuroprotective effect of bpV[pic] after ICH may be mediated through E2F1 and β-catenin signaling.

### BpV[pic] and PTEN Knockdown Reduces E2F1 Expression *in vitro*

**Figure 3:**
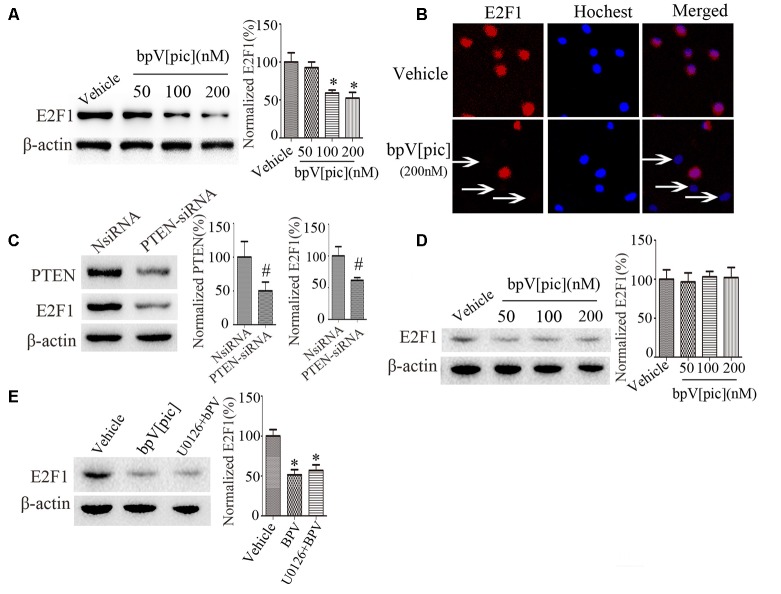
E2F1 was regulated by PTEN *in vitro*. **(A)** Western blot showed PTEN inhibitor bpV[pic] reduced E2F1 protein expression in a dose-dependent manner in primary cortical neurons **(B)** Immunofluorescent staining showed that E2F1 expression was decreased with bpV[pic] (200 nM) treatments in primary cortical neurons. **(C)** Western blot showed that PTEN siRNA decreased E2F1 protein level in primary cortical neurons. **(D)** In PTEN-deficient U251 cells, E2F1 was not decreased by PTEN suppression. **(E)** Western blot analysis showed that it did not change E2F1 protein levels when neurons were pretreated with extracellular signal-regulated kinase (ERK)1/2 inhibitor, which implied that bpV[pic] regulated E2F1 but not through ERK1/2 activation (*p < 0.05 compared to Vehicle, one-way ANOVA test, followed by Bonferroni *post hoc* test; ^#^*p < 0.05* compared to NsiRNA, Student’s *t*-test, *N* = 8).

To determine whether bpV[pic]-induced neuroprotection in ICH is mediated through E2F1 and β-catenin signaling, we examined the expression of E2F1 and β-catenin in primary cortical neurons and the PTEN-deficient human glioblastoma U251 cells after treatment with bpV[pic] or PTEN siRNA. Treatment with bpV[pic] (50, 100, 200 nM) or PTEN siRNA decreases E2F1 protein levels (Figures 3A,C). Immunofluorescence labeling also shows that bpV[pic] treatment reduces E2F1 protein expression (Figure 3B).

As the expression of E2F1 is not significantly changed in U251 cells that are PTEN-deficient (Figure 3D), these results indicate that regulation of E2F1 by bpV[pic] is dependent on PTEN. Our previous results reveal that bpV[pis] confers neuroprotection through suppressing PTEN and activating extracellular signal-regulated kinase (ERK)1/2 (Zhang et al., 2017), indicating that the inhibitory effect of bpV[pis] on PTEN is not specific. To test whether bpV[pic] regulates E2F1 through ERK1/2 activation, we treated cultured neurons with bpV[pic] (200 nM) with or without ERK1/2 inhibitor (U0126, 10 μM). The results show that the E2F1 protein levels are not altered when neurons are pretreated with ERK1/2 inhibitor (Figure 3E), implying that bpV[pic] regulates E2F1 independent of ERK1/2 activation.

### Knockdown of E2F1 Increases β-Catenin Expression in Cultured Cortical Neurons

**Figure 4:**
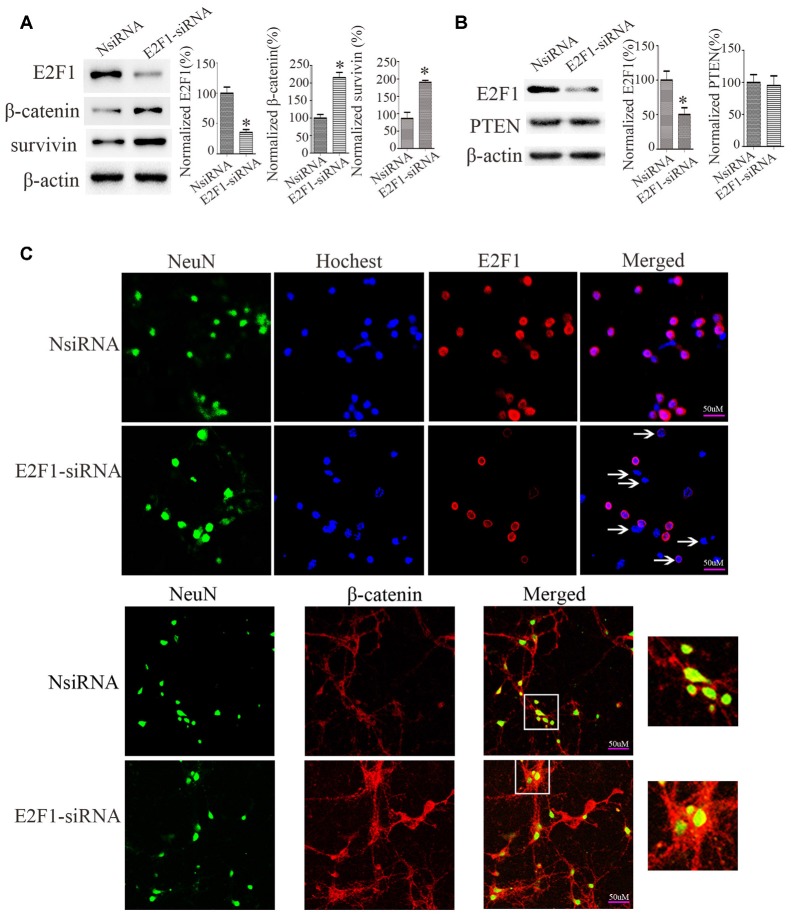
E2F1 knockdown upregulated β-catenin in primary cortical neurons. **(A)** Western blot showed β-catenin and its target gene survivin were increased when E2F1 was knocked down by E2F1 siRNA. **(B)** Western blot showed that PTEN was not changed significantly when E2F1 was knocked down by E2F1 siRNA. **(C)** Rat primary cortical neurons were cultured for 12 days, then treated with E2F1 siRNA for 24 h, and immunofluorescence was used to detect the expression of β-catenin. Results showed that protein levels of β-catenin, which is located in cytoplasm, nuclei, and axon were increased when E2F1 was knocked down in rat cortical neurons (*p < 0.05 compared to NsiRNA, Student’s *t*-test, *N* = 8).

To determine the relationship between E2F1 and β-catenin, we knocked down E2F1 expression in cortical neurons. The expressions of β-catenin and β-catenin-targeted gene survivin, a β-catenin activation marker (Jeong et al., 2014), were increased after knockdown of E2F1 (Figure 4A), but the protein level of PTEN did not change significantly (Figure 4B). To confirm this result, we conducted experiments in primary neuron cultures by immunofluorescence labeling and show that β-catenin expression was increased after knockdown of E2F1 (Figure 4C). These results indicate that E2F1 negatively regulates β-catenin expression in neurons.

### E2F1 Mediates PTEN Regulation of β-Catenin in Cortical Neurons

**Figure 5:**
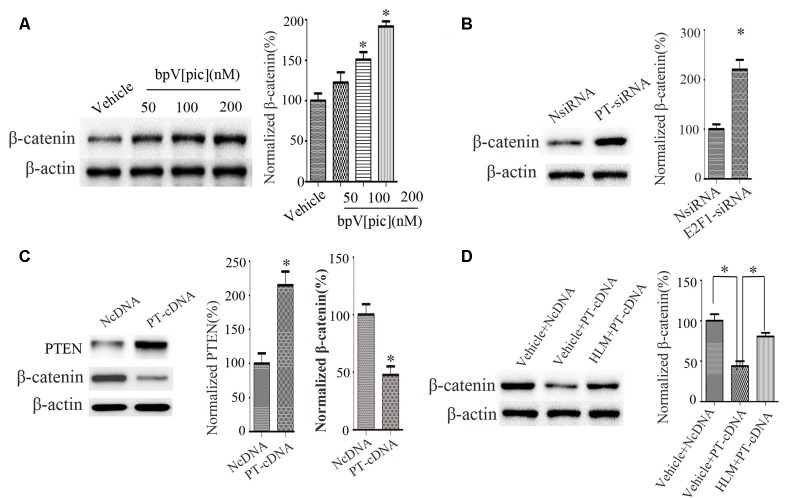
PTEN downregulated β-catenin, and this depended on E2F1 in primary cortical neurons. **(A)** Western blot showed that bpV[pic] increased β-catenin expression in a dose-dependent manner in primary cortical neurons. (*p < 0.05 compared to Vehicle, one-way ANOVA test, followed by Bonferroni *post hoc* test). **(B)** The expression of β-catenin was increased when PTEN siRNA was transfected in primary cortical neurons for 24 h (*p < 0.05 compared to NsiRNA, Student’s *t*-test). **(C)** The expression of β-catenin was decreased when PTEN was overexpressed in primary cortical neurons for 24 h (*p < 0.05 compared to NcDNA, Student’s *t*-test). **(D)** Western blot showed that E2F1 inhibitor HLM006474 attenuated the PTEN overexpression-induced downregulation of β-catenin when pretreated with HLM006474 for 12 h before overexpressing PTEN in neuronal cells (*p < 0.05, one-way ANOVA test, followed by Bonferroni *post hoc* test, *N* = 8; PT-cDNA, PTEN cDNA; PT-siRNA, PTEN siRNA).

We next examined the relationship between PTEN, E2F1, and β-catenin. We treated the cortical neurons with bpV[pic] (50, 100, 200 nM). We found that bpV[pic]-induced increase of β-catenin is concentration-dependent (Figure 5A). When we knocked down PTEN, the expression of β-catenin was increased (Figure 5B). On the contrary, overexpression of PTEN decreased the protein level of β-catenin (Figure 5C).

To investigate whether PTEN regulates β-catenin depending on E2F1, we pretreated neurons with E2F1 inhibitor HLM006474 (25 μM) for 12 h and then overexpressed PTEN for 24 h. The results show that HLM006474 attenuates the PTEN overexpression-induced downregulation of β-catenin (Figure 5D), suggesting that the regulation of β-catenin by PTEN is dependent on E2F1.

### BpV[pic] Prevents Hemin-Induced Neuronal Injury Through Downregulation of E2F1 and Upregulation of β-Catenin

**Figure 6:**
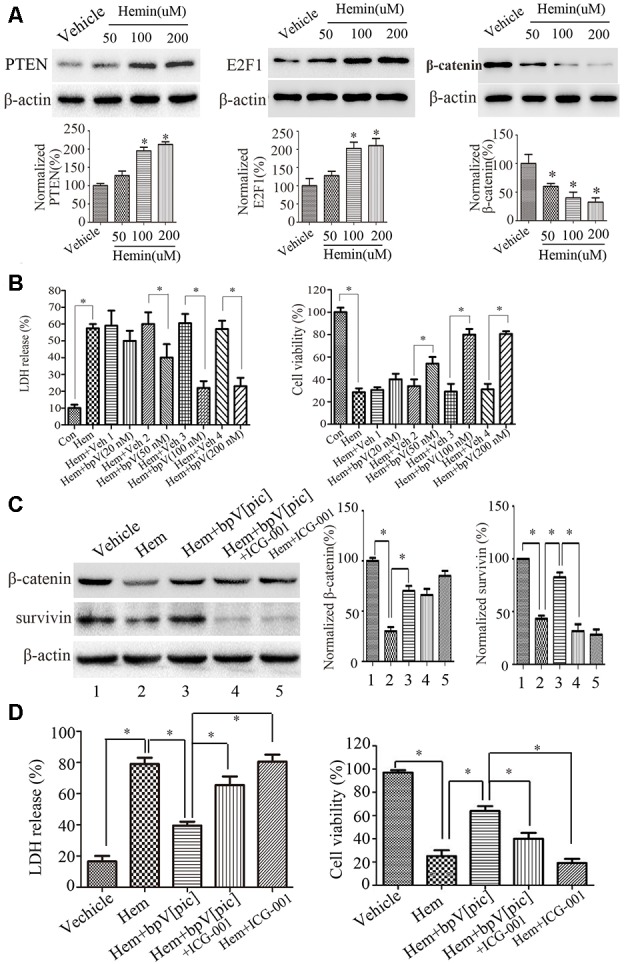
BpV[pic] prevented hemin-induced neuronal damage through the regulation of β-catenin expression and activity *via* PTEN/E2F1 pathway *in vitro*. **(A)** Western blot showed it increased the expression of PTEN and E2F1, decreased the expression of β-catenin concentration-dependently in hemin-treated cultured rat cortical neurons for 24 h (*p < 0.05 compared to Vehicle, one-way ANOVA test, followed by Bonferroni *post hoc* test, *N* = 8). **(B)** It decreased lactate dehydrogenase (LDH) release and increased cell viability dose-dependently 6 h after bpV[pic] (50–200 nM) administered in hemin-induced rat cortical neuron injuries (*p < 0.05, one-way ANOVA test, followed by Bonferroni *post hoc* test, *N* = 8). **(C)** Rat cortical neurons treated with bpV[pic] were administered with or without β-catenin inhibitor ICG-001. Western blot showed that bpV[pic] increased the levels of β-catenin and survivin (a β-catenin activation marker), which were decreased by hemin. Meanwhile, ICG-001 blocked the expression of survivin (*p < 0.05, one-way ANOVA test, followed by Bonferroni *post hoc* test, *N* = 8). **(D)** LDH and MTT assay showed that the protective effect of bpV[pic] against hemin-induced neurotoxicity was blocked by ICG-001 (*p < 0.05, one-way ANOVA test, followed by Bonferroni *post hoc* test, *N* = 8; Hem, Hemin; Veh, Vehicle; ICG-001, β-catenin inhibitor).

According to the findings above, we hypothesized that bpV[pic] exerts a neuroprotective effect through PTEN/E2F1/β-catenin pathway in rat ICH injury. Cultured rat cortical neurons were exposed to hemin or vehicle for 6 h with different concentrations (12.5, 25, 50, 100, 200 μM; He et al., 2010; Chang et al., 2011). The results show that with the increase of hemin concentration (50, 100, 200 μM), the release rate of LDH in rat cortical neurons is gradually increased, while the cell viability is decreased gradually (Supplementary Figure S2). At 24 h after hemin administration (50, 100, 200 μM), the expression levels of PTEN and E2F1 were increased, and the expression of β-catenin was decreased in a dose-dependent manner (Figure 6A). Based on these results, the concentration (100 μM) of hemin was selected in the following experiment. We treated the neuronal cultures with bpV[pic] (20, 50, 100, 200 nM) for 6 h at 30 min after hemin insult. We show that bpV[pic] (50, 100, 200 nM) reduces hemin-induced neuronal toxicity compared to vehicle groups (Figure 6B).

It is well-known that activation of β-catenin signaling results in neuroprotection (Jeong et al., 2014). To explore whether bpV[pic] reduces hemin-induced neuronal toxicity *via* activating β-catenin, rat cortical neurons treated with bpV[pic] were administered with or without β-catenin inhibitor ICG-001. We found that bpV[pic] increased the levels of β-catenin and survivin (a β-catenin activation marker), which were decreased by hemin (Figure 6C). Meanwhile, ICG-001 suppressed the expression of survivin (Figure 6C). The protective effect of bpV[pic] against hemin-induced neurotoxicity was blocked by ICG-001 (Figure 6D). Collectively, these data suggest that hemin may induce neurotoxicity *via* upregulation of PTEN and E2F1, contributing to downregulation of β-catenin, and that bpV[pic] may prevent hemin-induced neuronal damage through the regulation of PTEN/E2F1/β-catenin pathway.

### BpV[pic] Attenuates Brain Injury Through PTEN/E2F1/β-Catenin Pathway After Rat ICH Injury

**Figure 7:**
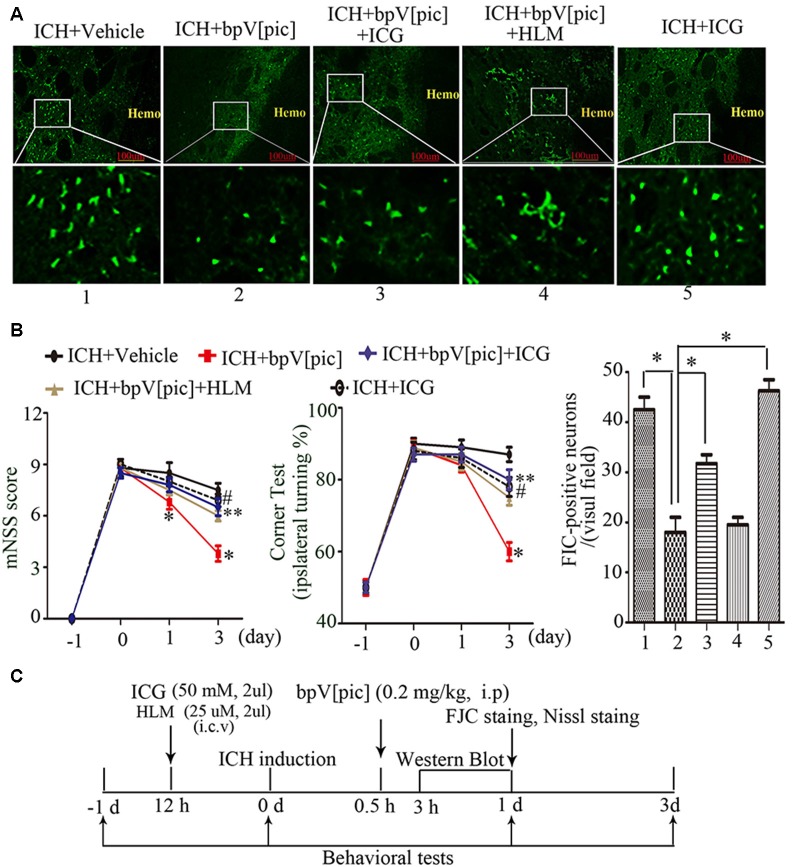
BpV[pic] attenuated brain injury through PTEN/E2F1/β-catenin pathway after rat experimental ICH. **(A)** Representative microphotographs of FJC-positive neurons in the peripheral tissues of hematoma after ICH. Compared with vehicle recipients, bpV[pic] reduced the FJC-positive neurons. ICG-001 blocked the protective effect of bpV[pic] against ICH-induced brain injuries (*P < 0.05, one-way ANOVA test, followed by Bonferroni *post hoc* test, *N* = 8). **(B)** Rats with bpV[pic] injection have lower scores of modified Neurological Severity Score (mNSS) test and corner test at days 1 and 3 after ICH. Rats preinjected with ICG-001 have higher scores of mNSS test and corner test at day 3 after ICH (*P < 0.05, ICH+bpV[pic] vs. ICH+Vehicle; **P < 0.05, ICH+bpV[pic]+ICG vs. ICH+bpV[pic]; ^#^*P* < 0.05, ICH+ICG vs. ICH+bpV[pic]; two-way ANOVA test, followed by Bonferroni *post hoc* test, *N* = 8). **(C)** A diagram shows the experimental procedures in ICH rats. ICH animals were subjected to three times of bpV[pic] intraperitoneal injection for the interval of 4 h 0.5 h after ICH induction. ICG-001 and HLM006474 were administered 12 h before ICH. FJC and Nissl staing were analyzed at 1 day after ICH. Behavioral tests were conducted 1 day before and 0, 1, 3 days after ICH induction (HLM, HLM006474; ICG, ICG-001).

To test whether bpV[pic] confers neuroprotection in ICH injury through PTEN/E2F1/β-catenin pathway, ICH rats were pretreated with HLM006474 [25 μM, 2 μl, intracerebroventricular injection (i.c.v.)] and/or ICG-001 (50 mM, 2 μl, i.c.v.) for 12 h and administered bpV[pic] [0.2 mg/kg, intraperitoneally (i.p.)] 30 min after ICH induction. Compared with vehicle recipients, administration of bpV[pic] reduced FJC-positive neurons in the perihematomal regions and reduced neurodeficits significantly at days 1 and 3 after ICH (Figures 7A,B). On the other hand, ICG-001 blocked the protective effect of bpV[pic] against ICH-induced brain injuries. These findings strongly indicated that PTEN inhibition by bpV[pic] was neuroprotective in rat experimental ICH *via* PTEN/E2F1/β-catenin pathway.

## Discussion

Hematoma enlargement and subsequent formation of brain edema induce cellular and molecular processes that cause neuronal death, thereby aggravating damage after ICH (Matsushita et al., 2000). Prospective treatments reducing neuronal death may provide a neuroprotective strategy for ICH patients. This study provides the evidence that bpV[pic], a PTEN inhibitor, attenuates ICH-induced brain injury in rats. BpV[pic] significantly reduced neurodeficits and neuron death around hematomas by injection of autologous blood. Results suggest that bpV[pic] has a potential therapeutic value for reducing ICH injury.

Mechanistic studies have shown the important role of E2F1 in cell apoptosis and proliferation. Despite many years of investigation, the role of E2F1 in regulating the fate of normal cells and cancer cells remains controversial. On the one hand, it is generally believed that activation of E2F1 plays a key role in driving normal cells into the cell cycle (Shan and Lee, 1994). On the other hand, it is also well-recognized that overexpression of E2F1 promotes apoptosis or growth arrest (Elliott et al., 2001). The latest study indicated that low levels of exogenous E2F1 promote proliferation, moderate levels induce G1, G2, and mitotic cell cycle arrest, and very high levels promote apoptosis (Shats et al., 2017). Interestingly, several lines of evidence implicated the roles of E2F members in cell death (Giovanni et al., 2000; Osuga et al., 2000; Park et al., 2000). It was reported that PI3K pathway selectively prevents E2F1-mediated activation of a subset of E2F1 target genes, including several apoptotic genes (Hallstrom et al., 2008). Deregulated levels of E2F1 are observed after ischemia (Osuga et al., 2000). Administration of a CDK inhibitor blocks the increase in E2F1 levels and dramatically reduces neuronal death by 80% after ischemia (Osuga et al., 2000). It also reports that decreased brain infarct following focal ischemia in mice lacks the transcription factor E2F1 (MacManus et al., 1999, 2003). Knockdown of E2F1 in cardiomyocytes inhibits necrotic cell death, and E2F1 knockout mice show reduced necrosis and myocardial infarct size upon ischemia/reperfusion (I/R; Wang et al., 2015). These results and our results imply that E2F1 is an important therapeutic target for the treatment of brain injury. In our study, it was demonstrated that E2F1 was increased distinctly after rat experimental ICH. At the same time, PTEN was upregulated significantly. When we administered bpV[pic] (the inhibitor of PTEN) or PTEN siRNA to suppress PTEN, E2F1was suppressed too. It made us to suppose that E2F1 was a target of PTEN, which was involved in neuronal injury after ICH. Our results confirm that PTEN regulates E2F1 expression, and the underlying mechanism may be that PTEN binds to E2F1 promoter region and interacts with E2F1 (Malaney et al., 2018).

Previous study showed that E2F1 represses β-catenin transcription and is antagonized by both pRB and CDK8 (Morris et al., 2008). E2F1 inhibits β-catenin activity *via* transcriptional antagonism and β-catenin degradation, and this inhibition contributes to E2F1-induced apoptosis (Morris et al., 2008). Taken together, we speculate that PTEN may regulate neuronal death after ICH through E2F1/β-catenin signaling pathway. Surprisingly, when the protein level of PTEN and E2F1 was changed after ICH, at the same time, β-catenin was decreased too. However, after suppression of PTEN, β-catenin was upregulated. To detect whether or not PTEN regulates β-catenin depending on E2F1, we overexpressed PTEN and knocked down E2F1 *in vitro*. The results showed that β-catenin was reduced upon overexpression of PTEN, but it did not change significantly with overexpression of PTEN and inhibiting E2F1 at the same time. From the results above, it can be concluded that PTEN downregulates β-catenin that relies on E2F1.

To investigate whether PTEN inhibition attenuates brain damage following ICH in rats *via* the PTEN/E2F1/β-catenin pathway, we performed a series of experiments both *in vivo* and *in vitro*. The results showed that bpV[pic] reduced the number of neuronal death and neurodeficits after ICH in rats while decreasing hemin-induced LDH release and increasing hemin-induced cell viability. However, ICG-001, the inhibitor of β-catenin, blocked the protective effect of bpV[pic] against ICH-induced brain injuries and hemin-induced neuron injuries.

Based on the above experimental results, this study first discovered and reported that PTEN/E2F1/β-catenin is a new signaling pathway involved in the development and prognosis of ICH in rats. It was also confirmed that the PTEN inhibitor bpV[pic] exerts neuroprotective effects after ICH in rats through the PTEN/E2F1/β-catenin signaling pathway. Although bpV[pic] is a commercially available PTEN inhibitor, it is indeed not specific to PTEN. We have previously demonstrated that bpV[pic] protects against ischemic neuronal death through PTEN inhibition and ERK1/2 activation (Liu et al., 2018). We reveal that the effect of bpV[pic] on ERK1/2 activation is independent of bpV[pic] inhibition on PTEN (Liu et al., 2018). To reveal the effect of PTEN inhibition on neuronal survival after ICH and hemin-induced injury in this study, we used PTEN-siRNA and PTEN cDNA in some of our experiments. We also combined the use of ERK1/2 inhibitor and bpV[pic] in some of the experiments to exclude the effect of ERK1/2 activation. Thus, while our results suggest that PTEN inhibition confers neuroprotection through E2F1 and β-catenin pathway, we cannot rule out the neuroprotective role of ERK1/2 activation induced by bpV[pic] in our experimental condition after ICH injury.

The recent studies show that miR-130a promotes neuronal growth in brain tissues in ICH rats by PTEN inhibition through PI3K/AKT pathway (Zhang et al., 2019). Recent studies also report that interleukin (IL)-10 mediates the polarization of microglia from M1 to M2 after ICH by increasing the expression of glycogen synthase kinase 3 beta (GSK3β), thereby inactivating PTEN in microglia (Zhou et al., 2017). The results imply that PTEN inhibition will help reduce brain damage after ICH, which is consistent with our results.

In our previous study, we described the neuroprotective effect of bpV(pis) that is designed by us. We show that bpV(pis) confers neuroprotection through suppressing PTEN and activating ERK1/2 (Liao et al., 2019). In this study, we used the commercially available PTEN inhibitor bpV[pic]. We investigated the protective effect of PTEN inhibition on lipid phosphatase function in rat ICH through E2F1/β-catenin pathway. However, according to other reports, GSK-3β is activated after ICH, which increases the phosphorylation of β-catenin serine/threonine residues, leading to a decrease in the stability of β-catenin and an increase in degradation, resulting in BBB destruction (Krafft et al., 2013). To date, the role of PTEN protein phosphatase function in β-catenin has not been reported in ICH. In the next step, we will further explore the role of PTEN’s dual function in ICH and seek more reasonable targets for clinical treatment.

Some limitations of this study should be considered. First, the mechanism of ICH is complicated. In addition to the hemin released by red blood cell destruction, iron ions, thrombin, immune factor release, BBB destruction, and so on are all ICH injury factors. As for the *in vitro* model, the administration of hemin to damage rat primary cortical neurons, because of the single damage factor, is not sufficient enough to truly reflect the pathophysiological changes of ICH. Second, collagenase-induced ICH model collagenase is a proteolytic enzyme that can damage BBB and brain to a greater extent than blood toxicity alone. Collagenase and autologous blood-induced ICH have different injury mechanisms and may result in different results; however, collagenase-induced cerebral hemorrhage has not been investigated in this study. Third, anti-inflammatory properties of PTEN inhibition were not evaluated in this study. Gaining this information is crucial and should be a priority in our future studies.

## Conclusion

This study provides evidence that PTEN inhibition by bpV[pic] attenuates hemorrhagic brain injury and hemin-induced neuronal injury. BpV[pic] significantly reduces neurological deficits and neuronal death in ICH injury. We demonstrate that PTEN/E2F1/β-catenin signal pathway plays a critical role in the rat model of ICH. BpV[pic] prevents hemin-induced neuronal damage. In contrast, β-catenin inhibitor ICG-001 promotes neuronal death in ICH rats and hemin-induced injury. Together, these results suggest that PTEN inhibition by bpV[pic] prevents neuronal damage through PTEN/E2F1/β-catenin pathway after ICH injury.

## Data Availability Statement

All datasets generated for this study are included in the article/Supplementary Material.

## Ethics Statement

The animal study was reviewed and approved by Committee on Animal Care and Ethics, Wuhan University School of Medicine.

## Author Contributions

DZ, X-PQ, and X-YL carried out ICH model and behavioral assessment. Z-FZ and QW participated in the design of the study and performed the data analysis. DZ, RL, and YL carried out cortical neuron culture, transfection, and immunofluorescence staining. JC performed the FJC staining. DZ and S-FC carried out Western blot and drafted the manuscript.

## Conflict of Interest

The authors declare that the research was conducted in the absence of any commercial or financial relationships that could be construed as a potential conflict of interest.

## Supplementary Material

FIGURE S1 Representative photomicrographies of striatum region around the hematoma containing Nissl positive neurons of each experimental group. It showed intact-appearing neurons in sham group while ICH group showed shrunken and cracked neurons indicating neurodegeneration 1 day after ICH induction. BpV[pic] was injection at 0.5 h after ICH for three times for the interval of 4 h at the dose of 0.2 mg/kg. Shrunken and intact neurons markedly decreased after bpV[pic] treated.

FIGURE S2 LDH assay and MTT assay of hemin-induced rat cortical neurons injuries at the different concentration. Rat cortical neurons cultured for 12 days, results showed LDH release increased while cell viability decreased concentration dependently 6 h after hemin treatment (**p* < 0.05, compared to DMSO, one-way ANOVA test, followed by Bonferroni *post hoc* test, *N* = 8).

## References

[B1] Chang C. F. Chen S. F. Lee T. S. Lee H. F. Chen S. F. Shyue S. K. (2011). Caveolin-1 deletion reduces early brain injury after experimental intracerebral hemorrhage. Am. J. Pathol. 178, 1749–1761. 10.1016/j.ajpath.2010.12.023 21435456 PMC3078460

[B2] Chang J. Mancuso M. R. Maier C. Liang X. Yuki K. Yang L.. (2017). Gpr124 is essential for blood-brain barrier integrity in central nervous system disease. Nat. Med. 23, 450–460. 10.1038/nm.4309 28288111 PMC5559385

[B3] Chang N. El-Hayek Y. H. Gomez E. Wan Q. (2007). Phosphatase PTEN in neuronal injury and brain disorders. Trends Neurosci. 30, 581–586. 10.1016/j.tins.2007.08.006 17959258

[B4] Chen J. Zhuang Y. Zhang Z. F. Wang S. Jin P. He C.. (2016). Glycine confers neuroprotection through microRNA-301a/PTEN signaling. Mol. brain 9:59. 10.1186/s13041-016-0241-3 27230112 PMC4880874

[B5] Denechaud P. D. Lopez-Mejia I. C. Giralt A. Lai Q. Blanchet E. Delacuisine B.. (2016). E2F1 mediates sustained lipogenesis and contributes to hepatic steatosis. J. Clin. Invest. 126, 137–150. 10.1172/jci81542 26619117 PMC4701565

[B6] Elliott M. J. Dong Y. B. Yang H. McMasters K. M. (2001). E2F-1 up-regulates c-Myc and p14(ARF) and induces apoptosis in colon cancer cells. Clin. Cancer Res. 7, 3590–3597. 11705881

[B7] Flower O. Smith M. (2011). The acute management of intracerebral hemorrhage. Curr. Opin. Crit. Care 17, 106–114. 10.1097/MCC.0b013e328342f823 21169826

[B8] Giovanni A. Keramaris E. Morris E. J. Hou S. T. O’Hare M. Dyson N.. (2000). E2F1 mediates death of B-amyloid-treated cortical neurons in a manner independent of p53 and dependent on bax and caspase 3. J. Biol. Chem. 275, 11553–11560. 10.1074/jbc.275.16.11553 10766769

[B9] Hallstrom T. C. Mori S. Nevins J. R. (2008). An E2F1-dependent gene expression program that determines the balance between proliferation and cell death. Cancer cell 13, 11–22. 10.1016/j.ccr.2007.11.031 18167336 PMC2243238

[B10] He Y. Hua Y. Lee J. Y. Liu W. Keep R. F. Wang M. M.. (2010). Brain alpha- and beta-globin expression after intracerebral hemorrhage. Transl. Stroke Res. 1, 48–56. 10.1007/s12975-009-0004-x 20563289 PMC2887669

[B11] Howitt J. Lackovic J. Low L. H. Naguib A. Macintyre A. Goh C. P.. (2012). Ndfip1 regulates nuclear Pten import *in vivo* to promote neuronal survival following cerebral ischemia. J. Cell Biol. 196, 29–36. 10.1083/jcb.201105009 22213801 PMC3255971

[B12] Hsu H. C. Liu Y. S. Tseng K. C. Tan B. C. Chen S. J. Chen H. C. (2014). LGR5 regulates survival through mitochondria-mediated apoptosis and by targeting the Wnt/beta-catenin signaling pathway in colorectal cancer cells. Cell. Signal. 26, 2333–2342. 10.1016/j.cellsig.2014.07.004 25025569

[B13] Hussaini S. M. Choi C. I. Cho C. H. Kim H. J. Jun H. Jang M. H. (2014). Wnt signaling in neuropsychiatric disorders: ties with adult hippocampal neurogenesis and behavior. Neurosci. Biobehav. Rev. 47, 369–383. 10.1016/j.neubiorev.2014.09.005 25263701 PMC4258146

[B14] Jeong J. K. Lee J. H. Moon J. H. Lee Y. J. Park S. Y. (2014). Melatonin-mediated beta-catenin activation protects neuron cells against prion protein-induced neurotoxicity. J. Pineal Res. 57, 427–434. 10.1111/jpi.12182 25251028

[B15] Jiang B. Li L. Chen Q. Tao Y. Yang L. Zhang B.. (2017). Role of glibenclamide in brain injury after intracerebral hemorrhage. Transl. Stroke Res. 8, 183–193. 10.1007/s12975-016-0506-2 27807801

[B16] Kononenko N. L. Classen G. A. Kuijpers M. Puchkov D. Maritzen T. Tempes A.. (2017). Retrograde transport of TrkB-containing autophagosomes *via* the adaptor AP-2 mediates neuronal complexity and prevents neurodegeneration. Nat. Commun. 8:14819. 10.1038/ncomms14819 28387218 PMC5385568

[B17] Krafft P. R. Caner B. Klebe D. Rolland W. B. Tang J. Zhang J. H. (2013). PHA-543613 preserves blood-brain barrier integrity after intracerebral hemorrhage in mice. Stroke 44, 1743–1747. 10.1161/strokeaha.111.000427 23613493 PMC3696522

[B18] Liao X. Y. Lei Y. Chen S. F. Cheng J. Zhao D. Zhang Z. F.. (2019). The neuroprotective effect of bisperoxovandium (pyridin-2-squaramide) in intracerebral hemorrhage. Drug Des. Devel. Ther. 13, 1957–1967. 10.2147/dddt.s204956 31354241 PMC6585412

[B19] Lin S. Yin Q. Zhong Q. Lv F. L. Zhou Y. Li J. Q.. (2012). Heme activates TLR4-mediated inflammatory injury via MyD88/TRIF signaling pathway in intracerebral hemorrhage. J. Neuroinflammation 9:46. 10.1186/1742-2094-9-46 22394415 PMC3344687

[B20] Liu B. Li L. Zhang Q. Chang N. Wang D. Shan Y.. (2010). Preservation of GABAA receptor function by PTEN inhibition protects against neuronal death in ischemic stroke. Stroke 41, 1018–1026. 10.1161/strokeaha.110.579011 20360540

[B21] Liu R. Tang J. C. Pan M. X. Zhuang Y. Zhang Y. Liao H. B.. (2018). ERK 1/2 activation mediates the neuroprotective effect of BpV(pic) in focal cerebral ischemia-reperfusion injury. Neurochem. Res. 43, 1424–1438. 10.1007/s11064-018-2558-z 29882124 PMC6006215

[B22] Ma L. Manaenko A. Ou Y. B. Shao A. W. Yang S. X. Zhang J. H. (2017). Bosutinib attenuates inflammation *via* inhibiting salt-inducible kinases in experimental model of intracerebral hemorrhage on mice. Stroke 48, 3108–3116. 10.1161/strokeaha.117.017681 29018127 PMC5679716

[B23] MacManus J. P. Jian M. Preston E. Rasquinha I. Webster J. Zurakowski B. (2003). Absence of the transcription factor E2F1 attenuates brain injury and improves behavior after focal ischemia in mice. J. Cereb. Blood Flow Metab. 23, 1020–1028. 10.1097/01.wcb.0000084249.20114.fa 12973018

[B24] MacManus J. P. Koch C. J. Jian M. Walker T. Zurakowski B. (1999). Decreased brain infarct following focal ischemia in mice lacking the transcription factor E2F1. Neuroreport 10, 2711–2714. 10.1097/00001756-199909090-00004 10511428

[B25] Maeda K. Takahashi N. Kobayashi Y. (2013). Roles of Wnt signals in bone resorption during physiological and pathological states. J. Mol. Med. 91, 15–23. 10.1007/s00109-012-0974-0 23111637

[B26] Maehama T. Dixon J. E. (1998). The tumor suppressor, PTEN/MMAC1, dephosphorylates the lipid second messenger, phosphatidylinositol 3,4,5-trisphosphate. J. Biol. Chem. 273, 13375–13378. 10.1074/jbc.273.22.13375 9593664

[B27] Malaney P. Palumbo E. Semidey-Hurtado J. Hardee J. Stanford K. Kathiriya J. J.. (2018). PTEN physically interacts with and regulates E2F1-mediated transcription in lung cancer. Cell Cycle 17, 947–962. 10.1080/15384101.2017.1388970 29108454 PMC6103743

[B28] Matsushita K. Meng W. Wang X. Asahi M. Asahi K. Moskowitz M. A.. (2000). Evidence for apoptosis after intercerebral hemorrhage in rat striatum. J. Cereb. Blood Flow Metab. 20, 396–404. 10.1097/00004647-200002000-00022 10698078

[B29] Milella M. Falcone I. Conciatori F. Cesta Incani U. Del Curatolo A. Inzerilli N.. (2015). PTEN: multiple functions in human malignant tumors. Front. Oncol. 5:24. 10.3389/fonc.2015.00024 25763354 PMC4329810

[B30] Morris E. J. Ji J. Y. Yang F. Di Stefano L. Herr A. Moon N. S.. (2008). E2F1 represses beta-catenin transcription and is antagonized by both pRB and CDK8. Nature 455, 552–556. 10.1038/nature07310 18794899 PMC3148807

[B31] Ning K. Pei L. Liao M. Liu B. Zhang Y. Jiang W.. (2004). Dual neuroprotective signaling mediated by downregulating two distinct phosphatase activities of PTEN. J. Neurosci. 24, 4052–4060. 10.1523/jneurosci.5449-03.2004 15102920 PMC6729419

[B32] Osuga H. Osuga S. Wang F. Fetni R. Hogan M. J. Slack R. S.. (2000). Cyclin-dependent kinases as a therapeutic target for stroke. Proc. Natl. Acad. Sci. U S A. 97, 10254–10259. 10.1073/pnas.170144197 10944192 PMC27851

[B33] Park D. S. Morris E. J. Bremner R. Keramaris E. Padmanabhan J. Rosenbaum M.. (2000). Involvement of retinoblastoma family members and E2F/DP complexes in the death of neurons evoked by DNA damage. J. Neurosci. 20, 3104–3114. 10.1523/jneurosci.20-09-03104.2000 10777774 PMC6773109

[B34] Poppy Roworth A. Ghari F. La Thangue N. B. (2015). To live or let die - complexity within the E2F1 pathway. Mol. Cell. Oncol. 2:e970480. 10.4161/23723548.2014.970480 27308406 PMC4905241

[B35] Ren H. Kong Y. Liu Z. Zang D. Yang X. Wood K.. (2018). Selective NLRP3 (pyrin domain-containing protein 3) inflammasome inhibitor reduces brain injury after intracerebral hemorrhage. Stroke 49, 184–192. 10.1161/strokeaha.117.018904 29212744 PMC5753818

[B36] Schmid A. C. Byrne R. D. Vilar R. Woscholski R. (2004). Bisperoxovanadium compounds are potent PTEN inhibitors. FEBS lett. 566, 35–38. 10.1016/j.febslet.2004.03.102 15147864

[B37] Shan B. Lee W. H. (1994). Deregulated expression of E2F-1 induces S-phase entry and leads to apoptosis. Mol. Cell. Biol. 14, 8166–8173. 10.1128/mcb.14.12.8166 7969153 PMC359355

[B38] Shan Y. Liu B. Li L. Chang N. Li L. Wang H.. (2009). Regulation of PINK1 by NR2B-containing NMDA receptors in ischemic neuronal injury. J. Neurochem. 111, 1149–1160. 10.1111/j.1471-4159.2009.06398.x 19780893

[B39] Shats I. Deng M. Davidovich A. Zhang C. Kwon J. S. Manandhar D.. (2017). Expression level is a key determinant of E2F1-mediated cell fate. Cell Death Differ. 24, 626–637. 10.1038/cdd.2017.12 28211871 PMC5384025

[B40] Sury M. D. Vorlet-Fawer L. Agarinis C. Yousefi S. Grandgirard D. Leib S. L.. (2011). Restoration of Akt activity by the bisperoxovanadium compound bpV(pic) attenuates hippocampal apoptosis in experimental neonatal pneumococcal meningitis. Neurobiol. Dis. 41, 201–208. 10.1016/j.nbd.2010.09.007 20875857 PMC2982859

[B41] Tiwari S. K. Agarwal S. Seth B. Yadav A. Nair S. Bhatnagar P.. (2014). Curcumin-loaded nanoparticles potently induce adult neurogenesis and reverse cognitive deficits in Alzheimer’s disease model *via* canonical Wnt/beta-catenin pathway. ACS Nano 8, 76–103. 10.1021/nn405077y 24467380

[B42] Wang K. An T. Zhou L. Y. Liu C. Y. Zhang X. J. Feng C.. (2015). E2F1-regulated miR-30b suppresses Cyclophilin D and protects heart from ischemia/reperfusion injury and necrotic cell death. Cell Death Differ. 22, 743–754. 10.1038/cdd.2014.165 25301066 PMC4392072

[B43] Xie Z. Huang L. Enkhjargal B. Reis C. Wan W. Tang J.. (2017). Intranasal administration of recombinant Netrin-1 attenuates neuronal apoptosis by activating DCC/APPL-1/AKT signaling pathway after subarachnoid hemorrhage in rats. Neuropharmacology 119, 123–133. 10.1016/j.neuropharm.2017.03.025 28347836 PMC5490977

[B44] Yamada K. M. Araki M. (2001). Tumor suppressor PTEN: modulator of cell signaling, growth, migration and apoptosis. J. Cell Sci. 114, 2375–2382. 11559746 10.1242/jcs.114.13.2375

[B45] Yang X. Sun J. Kim T. J. Kim Y. J. Ko S. B. Kim C. K.. (2018). Pretreatment with low-dose fimasartan ameliorates NLRP3 inflammasome-mediated neuroinflammation and brain injury after intracerebral hemorrhage. Exp. Neurol. 310, 22–32. 10.1016/j.expneurol.2018.08.013 30171865 PMC6203658

[B46] Zhang C. Y. Ren X. M. Li H. B. Wei W. Wang K. X. Li Y. M.. (2019). Effect of miR-130a on neuronal injury in rats with intracranial hemorrhage through PTEN/PI3K/AKT signaling pathway. Eur. Rev. Med. Pharmacol. Sci. 23, 4890–4897. 10.26355/eurrev_201906_18077 31210323

[B47] Zhang Q. G. Wu D. N. Han D. Zhang G. Y. (2007). Critical role of PTEN in the coupling between PI3K/Akt and JNK1/2 signaling in ischemic brain injury. FEBS Lett. 581, 495–505. 10.1016/j.febslet.2006.12.055 17239858

[B48] Zhang Z. F. Chen J. Han X. Zhang Y. Liao H. B. Lei R. X.. (2017). Bisperoxovandium (pyridin-2-squaramide) targets both PTEN and ERK1/2 to confer neuroprotection. Br. J. Pharmacol. 174, 641–656. 10.1111/bph.13727 28127755 PMC5368051

[B49] Zheng H. Chen C. Zhang J. Hu Z. (2016). Mechanism and therapy of brain edema after intracerebral hemorrhage. Cerebrovasc. Dis. 42, 155–169. 10.1159/000445170 27110940

[B50] Zheng M. Liao M. Cui T. Tian H. Fan D. S. Wan Q. (2012). Regulation of nuclear TDP-43 by NR2A-containing NMDA receptors and PTEN. J. Cell Sci. 125, 1556–1567. 10.1242/jcs.095729 22526419 PMC3336381

[B51] Zhou K. Zhong Q. Wang Y. C. Xiong X. Y. Meng Z. Y. Zhao T.. (2017). Regulatory T cells ameliorate intracerebral hemorrhage-induced inflammatory injury by modulating microglia/macrophage polarization through the IL-10/GSK3beta/PTEN axis. J. Cereb. Blood Flow Metab. 37, 967–979. 10.1177/0271678x16648712 27174997 PMC5363473

