# Supplementary figures and images for: PTEN Inhibition Protects Against Experimental Intracerebral Hemorrhage-Induced Brain Injury Through PTEN/E2F1/β-Catenin Pathway
Source: Front Mol Neurosci. 2019 Dec 5;12:281. doi: 10.3389/fnmol.2019.00281 (PMC6906195; doi:10.3389/fnmol.2019.00281)

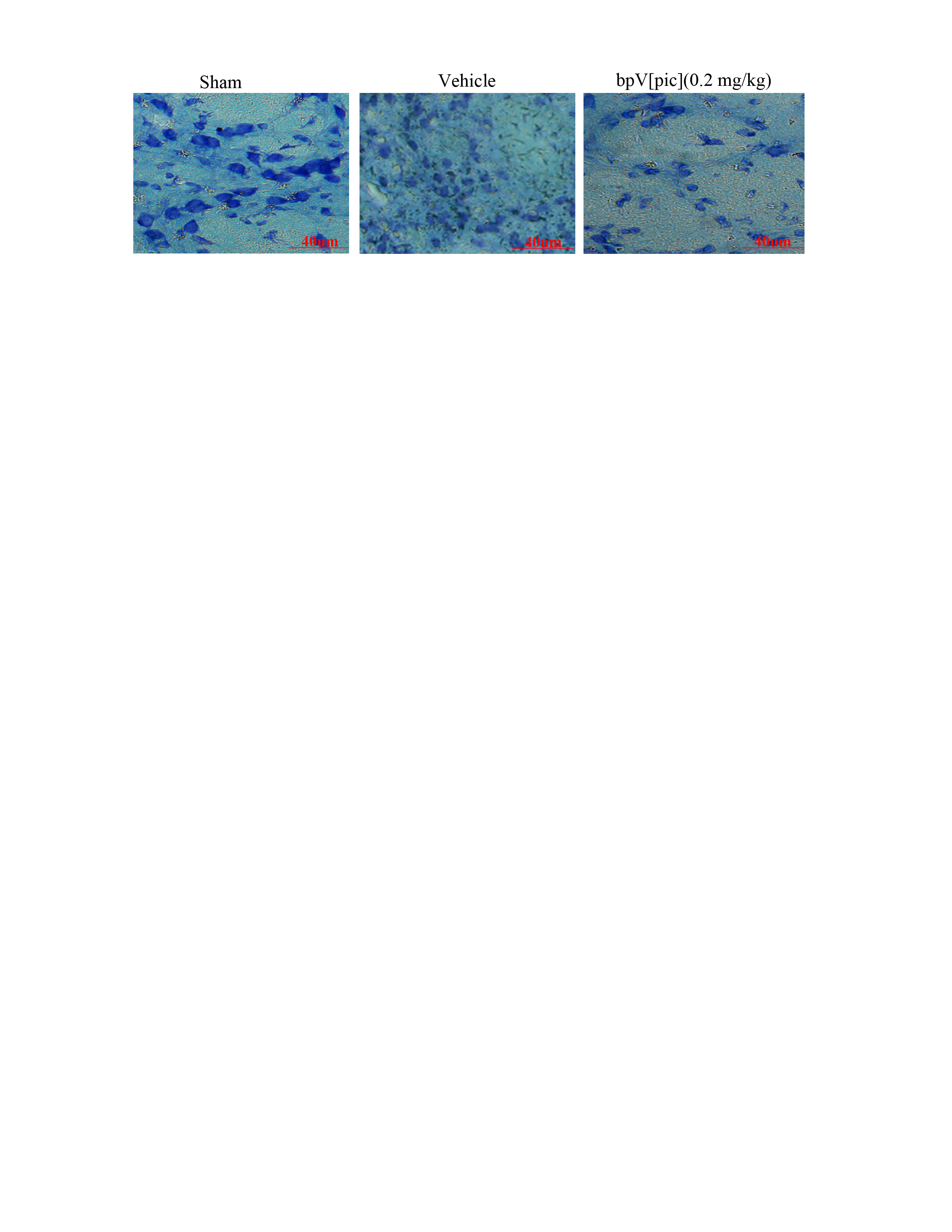

Supplement: FIGURE S1 — Representative photomicrographies of striatum region around the hematoma containing Nissl positive neurons of each experimental group. It showed intact-appearing neurons in sham group while ICH group showed shrunken and cracked neurons indicating neurodegeneration 1 day after ICH induction. BpV[pic] was injection at 0.5 h after ICH for three times for the interval of 4 h at the dose of 0.2 mg/kg. Shrunken and intact neurons markedly decreased after bpV[pic] treated. [file Image_1.jpeg]

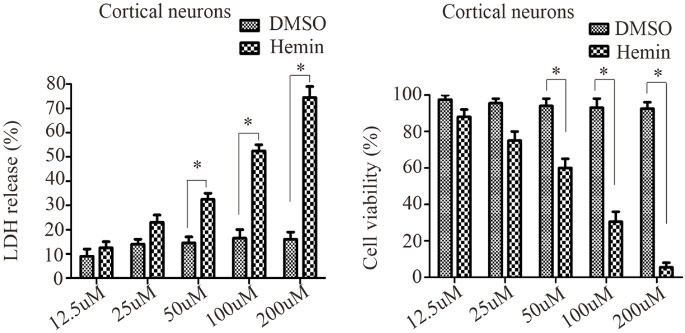

Supplement: FIGURE S2 — LDH assay and MTT assay of hemin-induced rat cortical neurons injuries at the different concentration. Rat cortical neurons cultured for 12 days, results showed LDH release increased while cell viability decreased concentration dependently 6 h after hemin treatment (*p < 0.05, compared to DMSO, one-way ANOVA test, followed by Bonferroni post hoc test, N = 8). [file Image_2.jpeg]
